# Application of ICA to realistically simulated ^1^H-MRS data

**DOI:** 10.1002/brb3.345

**Published:** 2015-04-25

**Authors:** Ravi Kalyanam, David Boutte, Kent E Hutchison, Vince D Calhoun

**Affiliations:** 1 The Mind Research NetworkAlbuquerque, New Mexico; 2 Department of ECE, University of New MexicoAlbuquerque, New Mexico; 3 Department of Psychology and Neuroscience, University of ColoradoBoulder, Colorado

**Keywords:** LCModel analysis, MRS ICA, proton spectroscopy, realistic simulations, spectral ICA

## Abstract

**Introduction:**

^1^H-MRS signals from brain tissues capture information on in vivo brain metabolism and neuronal biomarkers. This study aims to advance the use of independent component analysis (ICA) for spectroscopy data by objectively comparing the performance of ICA and LCModel in analyzing realistic data that mimics many of the known properties of in vivo data.

**Methods:**

This work identifies key features of in vivo ^1^H-MRS signals and presents methods to simulate realistic data, using a basis set of 12 metabolites typically found in the human brain. The realistic simulations provide a much needed ground truth to evaluate performances of various MRS analysis methods. ICA is applied to collectively analyze multiple realistic spectra and independent components identified with our generative model to obtain ICA estimates. These same data are also analyzed using LCModel and the comparisons between the ground-truth and the analysis estimates are presented. The study also investigates the potential impact of modeling inaccuracies by incorporating two sets of model resonances in simulations.

**Results:**

The simulated *fid* signals incorporating line broadening, noise, and residual water signal closely resemble the in vivo signals. Simulation analyses show that the resolution performances of both LCModel and ICA are not consistent across metabolites and that while ICA resolution can be improved for certain resonances, ICA is as effective as, or better than, LCModel in resolving most model resonances.

**Conclusion:**

The results show that ICA can be an effective tool in comparing multiple spectra and complements existing approaches for providing quantified estimates.

## Introduction

Proton (^1^H) magnetic resonance spectroscopy (MRS) offers a noninvasive window to observe in vivo brain metabolism. Though histopathology still remains the gold standard for tissue diagnosis, many studies have shown the importance of ^1^H-MRS in the understanding of major diseases. Identifying and quantifying the resonances captured in ^1^H-MRS data from brain tissues can reveal biomarkers for neuronal loss, disorder, or dysfunction (Horska and Barker 2010; Sturrock et al. 2010; Graff-Radford and Kantarci 2013; Groger et al. 2014). Even as MRS applications continued to grow, efforts on advancing methods to improve its performance are still ongoing. Scanner technology advances (Wiggins et al. 2006), new pulse sequences (Andronesi et al. 2010), and spectral editing (Bhattacharyya et al. 2007; Kaiser et al. 2008) are examples of some efforts to improve quality of acquired data, whereas, spectral quantification techniques and latent variable models such as independent component analysis (ICA) proposed in this manuscript are some of the postacquisition analysis techniques to improve MRS performance.

Acquired ^1^H-MRS data are typically noisy, and obtaining useful information from such data is a nontrivial exercise that is often time consuming and requires considerable spectroscopic expertise. Nonetheless, for spectroscopy to gain a meaningful role in clinical or research settings it is necessary to minimize the need for such expertise. One step in that direction is to decompose data into constituent components, and quantify components in terms of metabolites and their concentrations. Parameterizing MR data through quantifications remains the most popular way to compare multiple spectra. Most quantification methods, whether they work on the data in time domain, such as jMRUI (Naressi et al. 2001), or frequency domain, such as LCModel (Provencher 1993, 2001), employ prior information based on molecular structures of metabolites in estimating their concentrations.

Principal component analysis (PCA) (Stoyanova and Brown 2001) and independent component analysis (ICA) (Comon 1994) are statistical techniques optimized to identify structures in high-density data. They both make no assumptions on the shape of the structures underlying the data, and typically model data as linear combinations of such structures (sources or components). While PCA identifies mutually orthogonal structures in data and orders them by variance, ICA resolves data into a set of mutually independent structures. Our study aims to advance application of data-driven methods in the analysis of ^1^H-MRS data. Data-driven approaches can help address some common concerns associated with model based methods, such as vulnerability to modeling inaccuracies (Kreis 2004). The motivation for use of ICA comes from the hypotheses that observed neurochemical signals are linear mixtures of signals from metabolites whose concentrations can independently vary.

ICA has been employed in pediatric studies to identify and reject individual acquisitions affected by subject movement (de Nijs et al. 2009). It has also been applied to resolve simulated and real NMR data earlier in the classification of healthy and grades of tumor tissues in tumor studies (Ladroue et al. 2003; Pulkkinen et al. 2005). These studies applied ICA to analyze the starkly different spectra from healthy and pathology tissues and showed that ICA can be an effective, noninvasive alternative to intracranial biopsy in classifying tissue types. Our research work, rather than classification, focuses on applying ICA to parameterize ^1^H-MRS data from nonpathological tissues and yield parameters which can be useful in comparing multiple spectra. In our earlier study we demonstrated the robust performance of ICA in resolving ideal, noise-, and artifact-free-simulated data compared to LCModel, using two different spectral generative models, and observed the need for unambiguous evaluation of in vivo performances of the two methods (Kalyanam et al. 2013).

In this study, we examine the utility of ICA in analyzing more realistic ^1^H-MRS data and thus explore its applicability in analyzing in vivo datasets. In order to do that, it becomes necessary to design an artificial dataset that more closely mimics in vivo data, so that necessary *realistic* ground-truth is available to evaluate performances. To that effect, we extend simulations in our previous study by adding noise, line broadening, and other realistic ^1^H-MRS artifacts to the data. Some simulation experiments in tumor studies have also examined the effect of noise and artifacts on ICA's ability to *classify* spectra (Ladroue et al. 2003; Hao et al. 2009). Ours is the first study to *parameterize* spectra using ICA and compare its performance with that of LCModel. In this manuscript, we present comparative performances of ICA and LCModel in analyzing more realistic artificial datasets simulated with two different sets of basis spectra; in future, we plan to include other analyses, including jMRUI, in comparative evaluations.

### Theory

In this section, we briefly introduce two key methods used in our study, not commonly seen in most spectroscopy publications: ICA, a popular statistical technique with data-driven applications in a variety of fields, and Whittaker smoothing, a simple and robust smoothing technique developed earlier (Whittaker 1922; Macaulay 1931).

### Independent component analysis

ICA models data **X **= [**x**_**1**_ **x**_**2**_ **x**_**3**_**… x**_**n**_]^**T**^ as a linear combination of a set of independent sources **S **= [**s**_**1**_ **s**_**2**_ **s**_**3**_**… s**_**k**_]^**T**^ weighed by mixing coefficients **A **= [**a**_**1**_ **a**_**2**_ **a**_**3**_**… a**_**k**_]. For example, the linear construct for the observation **x**_**m**_ is given by the following equation:

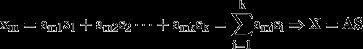


ICA can identify structures or components in data even when the shape of the distribution function of the data may be unknown. The goal of ICA is to resolve multivariate data **X** into linear combination of mutually independent components (ICs) **Y**; ICs are uniquely defined when at most one component is Gaussian (Comon 1994). ICA implementation involves iterative estimations of weights **W** that results in maximal mutual independence of components [**Y = WX].**

ICA implementations seek to maximize some measure of independence such as non-Gaussianity or mutual information: for example, *fast ICA* uses negentropy as its measure (Hyvarinen 1999), whereas *infomax* maximizes mutual information (Bell and Sejnowski 1995). In our analyses, we use the *infomax* algorithm to analyze data from our in vivo or simulation experiments, in the spectral domain. Prior to ICA, input data are centered, whitened and projected onto an orthonormal space in reduced dimensional space using PCA. We use a sigmoidal nonlinearity **y **= (1 + exp (−**u**))^−1^ to extract statistically maximally independent components 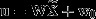 from the whitened data 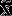, in an iterative manner, using stochastic gradient ascent optimization of weights **W**, where **W**_0_ is the bias. The learning rules to update weights at each iteration are given by the following equation:

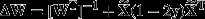
1

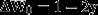
2

The algorithm converges and results in a consistent set of output components. Our previous study has demonstrated the applicability of ICA model to ^1^H-MRS problems validated using ideal, noise-free data (Kalyanam et al. 2013) and this study aims to evaluate it on more realistically simulated data.

### Whittaker smoothing

Smoothing is commonly carried out to suppress random data variations, to reveal data trending or to make meaningful predictions based on data. We utilize the Whittaker smoother, a penalized localized least-squares technique, to smooth ^1^H-MRS time series data to reveal the smooth-varying residual water signal that underlies the data. Recent works and implementations of this technique rekindled interest in this smoother and enabled many of its recent applications in many fields, including NMR (Eilers 2003). A brief introduction to this method is presented here for completeness.

Smoothing alters the value of each data point by taking its neighboring points into account and reduces variations between neighbors. Whittaker smoothing employs a penalized least-squares approach to provide a smooth fit to data, by simultaneously minimizing variations between neighbors (via a measure of roughness, R) and faithfulness of the smooth fit to the original data (via the fidelity or least-squares fit F); indeed, what is minimized is their linear sum, F + *h*R, where *h* is a positive number, called *smoothing parameter*, that determines the balance between fidelity and roughness (Weinert 2007). This single parameter provides continuous control of smoothing over the data and determines the degree of smoothing: larger *h* results in improved smoothing and reduced fit and, conversely, smaller *h* reduces smoothing, but improves fit.

The formulation presented can be applied to discrete data sampled at equal intervals, whereas an extension of the approach allows handling of data with nonuniform intervals or missing data. More details on the smoothing technique, its extensions and algorithms can be seen elsewhere (Eilers 2003). One concern with this technique is the subjectivity in the choice of smoothing parameter, but some studies have suggested statistical methods to set this parameter (Vilela et al. 2007; Lee and Cox 2010). Whittaker smoothing has been applied to NMR data, especially in spectral domain, earlier (Cobas et al. 2006). In such applications, typically signal-free regions are first identified by thresholding and noncontiguous signal-free regions are smoothed to obtain a smooth baseline.

## Materials and Methods

In this section, we describe the experimental conditions in which our in vivo data were acquired. We examine in vivo data to identify salient data features, and explore options to incorporate such features in simulations. We introduce our simulation basis-sets, ground-truth measures, and describe how we estimate parameters used in our simulations. We present how datasets of varying complexities are generated and provide details of the analysis techniques employed.

### In vivo experiment

Our in vivo ^1^H-MRS data come from a single, 12 cubic centimeters (cc) spectroscopic voxel in the anterior cingulate region of the human brain localized by point-resolved spectroscopy (PRESS), acquired with a TIM Trio 3-Tesla whole-body scanner (Siemens Medical Solutions, Erlangen, Germany; Syngo MR B15), using a 12-channel head coil receiver in conjunction with body coil excitation (TR/TE = 2 sec/40 msec, dwell time = 0.625 msec). The voxel is prescribed using a T1 scout image, and two sets of data, a water-suppressed data averaged from 192 *fid* signals and an unsuppressed water data averaged from 16 *fid* signals were obtained from the voxel. The acquired time series ^1^H-MRS data are stored in our data archive, for postacquisition analysis (Bockholt et al. 2010).

Data used in our analysis comes from 206 subjects (129 male, 76 female; ages 18–54), all patients, enrolled in three of our substance-abuse studies. All subjects provided informed consent to participate in these studies conducted at The Mind Research Network, Albuquerque, NM in accordance with protocols approved by the human research review committee of the University of New Mexico.

### LCModel analyses

LCModel analyses of in vivo data were carried out with no eddy-current correction, in the same 1.8–4.2 ppm analysis window used in our prior reports (Kalyanam et al. 2013; Yeo et al. 2013) and concentrations of all metabolites in the basis set estimated. In our in vivo analyses, we used acquired water signal as an internal reference to report absolute concentrations of metabolites (Soher et al. 1996). Suspect bad quality spectra, based on LCModel reported full-width half-maximum (*fwhm*), signal-to-noise ratio (S/N), and other quality considerations detailed in our earlier report were excluded and concentration estimates from the rest of in vivo data were used as ground-truth in our simulation experiments (Kalyanam et al. 2013). Our simulated data of varying complexities were also analyzed with the same settings; however, the metabolites concentrations are estimated relative to total-creatine. All data were analyzed in a consistent manner through use of a custom shell script.

### Simulation experiments

**Figure 1:**
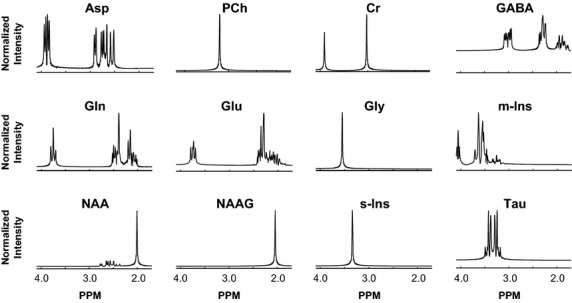
Model resonances of neurochemicals in the basis set: Real parts of LCModel basis spectra, intensity normalized, are shown in 1.8–4.2 ppm analysis window. Notice the varied spectral profiles of the model resonances.

In our simulations we use a basis set of 12 neurochemicals found in human brain and typically reported in LCModel quantification of an in vivo spectrum: aspartate (Asp), creatine (Cr), *γ*-amino butyric acid (GABA), glucose (Glc), glutamine (Gln), glutamate (Glu), myo-inositol (m-Ins), N-acetyl aspartate (NAA), N-acetylaspartylglutamate (NAAG), phosphocholine (PCh), scyllo-inositol (s-Ins), and taurine (Tau). Composition of basis set was chosen so that the model resonances of the neurochemicals comprise some singlet peaks, some multiplet peaks and some resonances which partially overlap with other resonances. We use two sets of models for each metabolite, one obtained from LCModel and another simulated using GAVA (Soher et al. 2007) and, just as in vivo data, model data were also saved as time series signals. A diverse set (see LCModel spectra of basis metabolites in Fig.1) allows us to establish the limitations and/or advantages of the analysis techniques based on the statistical properties of the model resonances.

**Figure 2:**
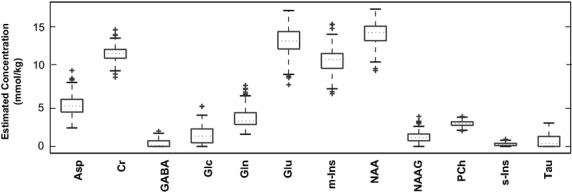
LCModel estimated in vivo metabolites concentrations: Box plots show distributions of metabolites concentrations from 206 subjects' in vivo data. We use these as ground-truth mixing coefficients in our simulation experiments. Notice the two-orders of magnitude difference between the median values of NAA and GABA.

Metabolite concentrations reported by LCModel analyses of a 206 subject in vivo dataset served as ground-truth mixing coefficients used to mix model resonances and obtain simulated datasets. Notice the considerable variability in the estimates of the metabolites (see Fig.2). While some metabolites (NAA, Cr, m-Ins Glu, Gln) are strongly present in all of the in vivo spectral data, metabolites like GABA and s-Ins are sparsely present, existing in far fewer spectral data. To generate the model data, we used in vivo estimates of total-creatine for Cr, total choline for PCh in addition to using Glc estimates for Gly. Noise- and artifact-free-simulated data are readily constructed by mixing basis model data using the 206 sets of mixing coefficients. Two sets of simulated data were generated, one using LCModel bases and other using GAVA bases.

### Data characterization

It is the intent of this study to significantly extend the noise- and artifact-free simulations described above, and also reported in our previous work, to obtain *realistic* simulated data (Kalyanam et al. 2013). To accomplish that, we closely examine the in vivo data, which is an average *fid* signal from multiple acquisitions. Our interest is in water-suppressed data, as suppressing the dominant signal from the water protons allows detection of weaker signals from the metabolite protons. In addition to contributions from metabolite protons of interest, such a signal also includes contributions from unsuppressed (residual) water protons, subject motion during scan, receiver noise, etc. Other confounding aspects are alterations of metabolite signal intensities due to magnetization transfer effects (Dreher et al. 1994) and nuclear Overhauser effects associated with selective (water) signal suppression. We present some salient characteristics of in vivo data and how they are incorporated into our simulations.

#### Residual water

**Figure 3:**
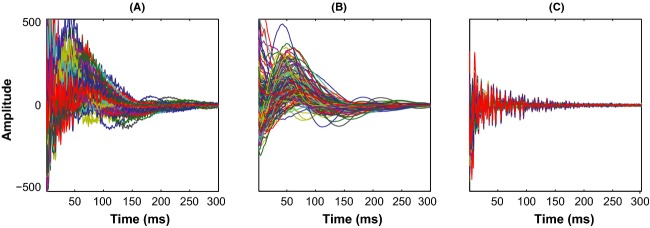
Residual water signal underlying time series signal: (A) shows real parts of in vivo *fid* signal from 206 subjects and (B) shows the residual water signal extracted by smoothing the time series signal. Notice how well *fid* signals show up after the removal of residual water (C).

It is common in some in vivo experiments to have limited water suppression and leave in some residual water signal, to allow for coherent averaging of data (Ernst and Li 2011). Subject motion during the long ^1^H-MRS scan acquisitions also can cause poor water suppression (Keating et al. 2010). The residual water signal, which can vary considerably from one scan to other, is a hard to model nonanalytic signal that typically appears as a slow-varying baseline in the time series data. Figure3A shows the dominating presence of residual water signal in our in vivo data. Smooth, slow-varying baselines appear as sharp, low-frequency peaks in the spectral domain, resulting in distortions that considerably affect estimations of resonances near water peak, and influence estimations of all other resonances through its influence on spectral baseline.

In ^1^H-MRS analyses it is necessary to reliably remove the residual water signal; not surprisingly, removal of residual water is a common step in most quantitation methods (Poullet et al. 2008). Some common techniques to remove residual water include use of Hankel singular value decomposition (SVD) or its many optimized avatars (Pijnappel et al. 1992), convolution with sine-bell filter (Drost et al. 2002), or maximum-phase finite impulse response filters (Sundin et al. 1999). While some use parametric modeling to account for the residual water signal, LCModel handles residual water by restricting the analysis window, simulating a *negative creatine CH*_*2*_ singlet (-CrCH_2_, 3.94 ppm) and treating the tail of residual water signal as part of its model-free baseline (Provencher 2001).

In our analysis, we smooth the time series data to eliminate signals from metabolites and random data variations to uncover the underlying smooth residual water signal. Though we used one common smoothing parameter to work on all in vivo data, it is seen effective in extracting baseline in most cases; in cases when residual water signal is very large, some baseline is still seen, but is vastly diminished (see Fig.3C). The extracted residual water signals serve us well to provide necessary complexity in our realistic simulations experiment. Another observation to be noted here is that, unlike Whittaker's original work which used three nearest neighbors in smoothing, we considered only *one* nearest neighbor, because such a choice results in a straight line at maximum smoothing, which preserves the spectral characteristics of the data.

#### Spectral broadening

Once the smooth baselines of time series data are removed, other signal features are more clearly revealed. Figure3C shows that the *fid* signals decay quickly and descend to noise level, within one-half of data points. As the spectral characteristics of a decaying, oscillatory signal, in time domain, is the convolution of the spectral characteristics of the oscillatory signal with that of the decay signal, the decay in time domain leads to spectral broadening. The nature of decay determines spectral line-shape and *fwhm* of the resonance peak; a natural (or exponential) decay results in Lagrangian line-shape, whereas Gaussian decay leads to a Gaussian line-shape. ^1^H-MRS *fid* signal is often modeled as natural or Gaussian decay function weighing sum of oscillatory signals. Some studies have modeled decay as a Voigt function, which is a combination of natural and Gaussian decay functions (Marshall et al. 1997; Gillies et al. 2006).

In order to incorporate spectral broadening for use in simulations, we explored the decay model supported by our in vivo data. We applied a Voigt fit to the absolute values of the time series data and examined decay-rate-constants of the Gaussian and Lagrangian components. We observed that, whereas Voigt function fitted *raw* time series data, as acquired, better than individual Lagrangian or Gaussian components, upon de-trending the data by removing the smooth baselines, the Lagrangian component alone fitted the data sufficiently well and the Gaussian component did not add much value. We therefore decided to use exponential weighting functions to broaden data in our simulations.

#### Noise

The *fid* signal in Fig.3C also clearly shows that, when the *fid* is fully decayed, its intensity varies randomly around some baseline. The random, zero mean fluctuations are attributed to receiver thermal noise and usually modeled as zero mean, unit-norm, white Gaussian noise. Though such noise exists even when the *fid* is strong, it is less visible when the signal is strong in the initial phases of the *fid* signal. The white noise model also sufficiently accounts for other uncorrelated sources of noise, such as, limited subject movement during scan, scan-to-scan variations in voxel positioning, field inhomogeneity, water suppression, etc. In our simulations we add white Gaussian noise to data at signal-to-noise ratio (S/N) levels estimated from our in vivo data.

### Realistic simulations

Spectral broadening is incorporated by multiplying each noise- and artifact-free-simulated time series signal with an exponentially decaying weighting function. The decay time constant, obtained from *fwhm* estimates from corresponding in vivo data, varies from one signal to other. A- fairly accurate estimate of *fwhm* for each spectrum was obtained from the resonance of its NAA's acetyl moiety by-estimating *fwhm* from the frequencies corresponding to its two half-maxima points. Our ability to obtain well-resolved *fwhm* estimations allows us to exercise fine control over spectral broadening.

White Gaussian noise is added to simulated data at S/N levels estimated from in vivo data. As a decaying *fid* signal acquired long after signal decayed to noise readily lends itself to S/N estimations, we used time series data to estimate the level of noise. As the initial data points of a *fid* are largely ‘signal’ and its final data points are largely noise, S/N is estimated as the ratio of variances of initial and final data points. In case of in vivo data, the presence of residual water can affect estimation accuracy, so it becomes necessary to remove fid's baseline beforehand.

Residual water signals are obtained by smoothing of in vivo data; the addition of residual water signal that varies from one in vivo data to other brings a touch of reality to the simulations experiment. Simulated datasets of varying complexities are generated by incorporating one or more of the artifacts/noise as described. Other in vivo data features, such as, phasing, or effects of subject movement during scan were not included in simulations, but will be important topics in our future work. All simulated datasets are analytical time series data; for use with LCModel, each individual data are saved in separate files, in a format consistent with in vivo data.

### ICA analyses

We applied ICA to analyze data in the spectral domain and simulated datasets of varying complexities were individually analyzed. As simulated data are in time domain, these data need to be converted into frequency domain using discrete Fourier transform (FFT) first, and depending on the complexity, may also require some degree of preprocessing, to prepare the data for ICA analysis. To avoid the paucity of data points which may obscure visibility of some fine spectral patterns and improve apparent resolution of the spectra, the time series data were zero-filled to double vector lengths of data. The data were then transformed to frequency domain, and *real* parts of the data, in the analysis window (1.8–4.2 ppm), were used in subsequent analysis. As the dispersive nature of imaginary part of the spectra degraded the resolution performance, we utilized the absorptive, real parts of data which contain most spectral energy in our analyses.

Input data were centered, whitened, decomposed using SVD, and dimension reduced prior to ICA analysis. The number of significant components retained varied based on simulation complexity, but, in general, are determined by the rank of the matrix or minimum descriptor length (Ojanen et al. 2004). Multiple runs of ICA analysis were performed on the dimension reduced data to ensure consistent set of components are resolved. Components across multiple runs were matched to one another and averaged, to yield ICs, which were then compared to generative basis spectra to identify and/or associate ICs with model resonances. The extracted ICs are inherently narrow and can resemble the narrow, generative model resonances. We call the Pearson correlation coefficient of a model resonance and its matching component as the *spectral correlation score*, a measure of how well ICA resolves that resonance.

Estimates of mixing coefficients, interchangeably called ICA estimates or weights, are obtained by regression analysis of each spectrum with the extracted ICs. If the analyzed data are not broadened, this approach works well. However, if the data are generated by mixing broadened bases or in vivo, the extracted ICs also need broadening, before applying the regression. We estimate *fwhm* of the spectrum as described before, then exponentially broaden ICs based on the spectral *fwhm*, and apply regression to estimate ICA weights. Just as LCModel provides estimate for each model resonance in its basis set, ICA provides an estimate for each IC. When an IC is identified with a model resonance based on spectral correlation, its corresponding ICA estimates are considered comparable to LCModel estimates for that resonance. In simulation analyses, LCModel and ICA estimates from spectral dataset, can be compared to the ground-truth; The Pearson correlation coefficient of the estimates to the ground-truth, called, *estimates correlation score* is a measure of how well an estimate tracks ground-truth.

## Results

In this section, we first show the set of basis spectra and ground-truth mixing coefficients used in our simulations. Then we illustrate salient in vivo data features incorporated in our simulations and how corresponding parameters are estimated; typical realistic simulated data presented and compared with in vivo. Next, we present the results from ICA and LCModel analyses of different simulated datasets to see how the two methods compare in analyzing imperfect data with realistic variations and features.

### Realistic simulations

Figure1 shows real parts of LCModel basis spectra of the low molecular weight metabolites in our basis set; only resonances within the 1.8–4.2 ppm analysis window are shown. Model spectra are based on prior information from quality in vitro experiments performed at high fields and/or derived from information on molecular structures and physical understanding. Notice the diverse spectral profiles of the basis set metabolites. The box plots in Fig.2 show the distributions of metabolites concentrations obtained from the LCModel analysis of our 206-subjects' single voxel in vivo data. These concentration estimates were used to mix model spectra in obtaining simulated data.

Figure3A shows the acquired *fid* signals from our 206-subjects' single voxel in vivo data. The dominant residual water signal present in the time series signal is extracted using Whittaker smoothing and shown in Fig.3B. Figure3C shows the residuary signal after the removal of smooth water signal from the time series data. This flat residuary signal clearly shows that the signal decays into noise as time progresses. As in vivo ^1^H-MRS *fid* signals decay quickly, we show the first third of the data points as the remainder of the data points are noisy and uninteresting.

**Figure 4:**
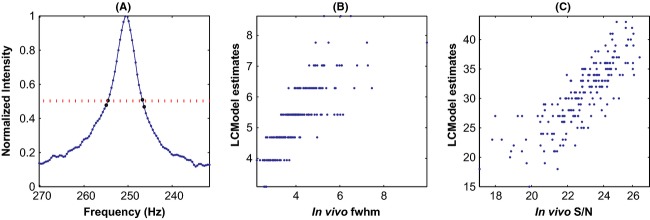
Estimation of *fwhm* and scatter plots of estimates: (A) shows acetyl moiety peak of a typical in vivo spectrum and highlights pair of data points on either side of each half-maximum; the frequencies corresponding to each pair is extrapolated to estimate *fwhm*. The scatter plot of our estimates of *fwhm* against LCModel estimates in (B) show that while LCModel estimates are discrete, our estimates are continuous. Tight scatter of S/N estimates shown in (C) validates both the estimation approaches.

Figure4A illustrates how we estimate *fwhm* from in vivo spectra. NAA's acetyl moiety peak is shown and the two pairs of data points nearby the half-maxima points are highlighted. The frequencies corresponding to half-maxima points are obtained by interpolation of the frequencies of highlighted data points and the difference between the frequencies gives the *fwhm* of the peak. The scatter plots in Fig.4B shows how our estimates of *fwhm* compares to the estimates from LCModel. Notice our estimates of *fwhm* are continuous compared to LCModel estimates which are quantized and discrete. Figure4C shows the scatter plot of our S/N estimates against those of LCModel. As mentioned previously, we estimate S/N from time domain data where as LCModel estimates it from data in spectral domain. The tight scatter between the estimates from two different approaches validates both the methods.

**Figure 5:**
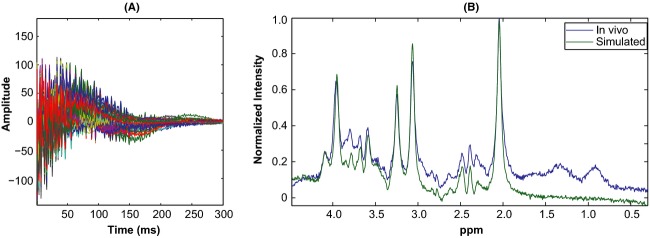
Realistic simulations: Real parts of 206 simulated time series signals shown compare well with corresponding in vivo signals shown in Fig.3A. Also shown is a typical in vivo spectrum and its corresponding simulated spectrum constructed using estimates from that spectrum and LCModel basis set. Notice how well the simulated spectrum resembles in vivo, despite visible differences due to noninclusion of lipids, lactates, and macromolecule resonances in the model.

Figure5 shows simulated data that incorporates line broadening, noise, and residual water signal; notice how well the simulated *fid* signals in Fig.5A resemble the in vivo signals in Fig.3A. Figure5B shows a typical in vivo spectrum and the simulated spectrum generated with signals/parameters estimated from that spectrum. The spectra are presented in a wider window to illustrate that the simulated spectrum is fairly realistic and complex, and differs from the in vivo primarily on resonances and features that are not included in the simulation model (lactates, lipids and macromolecules).

### Simulation analyses

Table 2010 illustrates the effect of zero mean, unit-norm Gaussian noise on ICA and LCModel analysis performances in the absence of spectral broadening or residual water signal. Table 2010A presents estimates correlation scores from the analyses of data generated using LCModel bases, whereas Table 2010B shows results from the analyses of data generated using GAVA bases. Each table presents estimates correlation scores from the analyses of noisy data as well as from ideal, noise-free data so that the effects of noise are readily seen.

**Table 1:** Effects of Gaussian noise on Analysis performances: Results from LCModel & ICA analyses of data generated with narrow LCModel/GAVA bases, with and without noise, shown. Ground-truth correlations of both LCModel and ICA estimates are high, and show little performance degradation due to noise. LCModel does not find Asp resonance in GAVA generated data, due to modeling differences, though ICA had no issue in resolving the metabolite's model resonance. (A) Results from analyses of data generated with LCModel bases; (B) Results from analyses of data generated with GAVA bases

	Asp	Cr	GABA	Glc	Gln	Glu	m-Ins	NAA	NAAG	PCh	s-Ins	Tau
(A)												
Ideal data, LCM analysis	1.000	1.000	0.998	0.999	1.000	0.999	1.000	1.000	1.000	1.000	1.000	0.999
Noisy data, LCM analysis	0.983	0.997	0.969	0.986	0.993	0.985	0.994	0.998	0.995	0.997	0.995	0.979
Ideal data, ICA analysis	0.997	1.000	0.995	0.991	0.996	0.985	0.999	1.000	0.998	0.998	0.990	0.917
Noisy data, ICA analysis	0.986	0.999	0.968	0.981	0.991	0.981	0.997	0.999	0.997	0.997	0.984	0.960

	Asp	Cr	GABA	Gly	Gln	Glu	m-Ins	NAA	NAAG	PCh	s-Ins	Tau
(B)												
Ideal data, LCM analysis	0	0.988	0.932	–	0.990	0.975	0.806	0.990	0.790	0.978	0.935	0.982
Noisy data, LCM analysis	0	0.987	0.906	–	0.986	0.970	0.811	0.988	0.783	0.975	0.929	0.978
Ideal data, ICA analysis	0.996	1.000	0.976	0.867	1.000	0.982	1.000	1.000	0.977	0.999	0.969	0.999
Noisy data, ICA analysis	0.985	0.999	0.754	0.869	0.992	0.989	0.996	0.999	0.969	0.998	0.968	0.995

Table 1995 presents the estimates correlation scores from ICA and LCModel analyses of simulated data that incorporates spectral broadening, in addition to noise; the results from the analyses of datasets generated with LCModel and GAVA bases are presented. To ease comparing the two methods, some ICA estimates are marked up: those bold, in green show resonances which ICA resolves better, and those bold, italicized in red are resolved better by LCModel. Table 2007 shows similar results from the analyses of simulated data that incorporates residual water, line broadening and noise; again, the results from LCModel and GAVA simulated data are shown.

**Table 2:** Effects of line broadening and noise on performance: Results from ICA and LCModel analyses of data generated with exponentially broadened bases, with additive white Gaussian noise shown. Clearly, broadening causes degradation in the performances of both methods, and some metabolites are better estimated than others

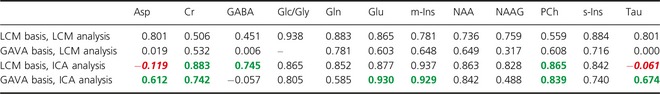

**Table 3:** Effects of line broadening, noise, and residual water signal on performance: Results from ICA and LCModel analyses of data generated with exponentially broadened bases, with additive white Gaussian noise and confounding residual water signal shown. Both methods are robust to residual water signal; little performance impact seen

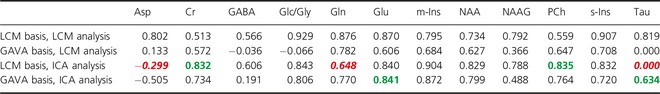

## Discussion

Results from our simulation experiments show that ICA, just as LCModel, resolves multivariate data and identifies underlying structures, traceable to generative model, and that ICA estimates correlate well with simulation ground-truth. Both LCModel and ICA offer comparable performances for many model resonances; ICA outperforms LCModel in resolving few resonances, but its performance in resolving some weak resonances needs improvement (e.g., ‘Asp’ and ‘Tau’).

Table 2010 shows that both LCModel and ICA improved their *ideal* data performance compared to our prior results (Kalyanam et al. 2013). This is a direct consequence of the recent improvements made in our simulated data generation. Though we still use the same set of models, GAVA model resonances were slightly broadened so that their line-widths are comparable to LCModel resonances; additionally, data mixing is carried out in time domain, unlike in spectral domain as before. These changes reduced differences between the two generative models and improved LCModel performance in resolving GAVA resonances. Nonetheless, LCModel's inability to resolve ‘Asp’ continues to remind us about the effect of modeling inaccuracy, when actual ground truth deviates from the assumed model. Meanwhile, ICA, which does not assume underlying data structures, offers comparable performance in analyzing LCModel simulated data and also resolves ‘Asp’ and ‘Gly’ resonances from GAVA simulated data. The results also reveal that ICA and LCModel performances are barely affected by the addition of Gaussian noise.

Table 1995 reveals the sensitivity of LCModel and ICA to cope with some realistic data features; not surprisingly, their performances have degraded when analyzing data that incorporates both spectral broadening and noise. We see that, in this case, LCModel could not resolve ‘GABA’ and ‘Tau’, in addition to ‘Asp’, from GAVA simulated data, whereas it resolved other resonances as well or only slightly poorly compared to LCModel simulated data. Though ICA does not resolve ‘Asp’ and ‘Tau’ resonances from LCModel data, it clearly outperforms LCModel in resolving singlet resonances. Poor performance of LCModel in resolving ‘Cr’ can be concern, especially if ‘Cr’ is used as the reference metabolite in relative concentration estimates. Table 1995 results show that though the presence of residual water signal lowers performance measures, the degradation is not as dramatic.

It is interesting to see that, except for ‘Asp’ and ‘Tau’, ICA is as effective as or better than LCModel in resolving model resonances from realistic simulated data. The resolution performance of ICA, just as LCModel's, varies from one resonance to other, based on the statistical properties of a model resonance and its corresponding concentration estimates (ground-truth). Though ICA's ability to resolve ‘Asp’ or ‘Tau’ is a cause for concern, its overall performance cannot be dismissed, given LCModel estimates are also not consistent across metabolites. These results demonstrate that ICA can play a role in analyzing ^1^H-MRS data; clearly, it cannot replace single-spectrum analysis methods such as LCModel, but can be very useful in comparing multiple spectra. We are working to further its performance by incorporating prior data for metabolites of concern and by developing preprocessing methods to account for some realistic data features.

## Conclusion

We have discussed our rationale for why realistic simulations are useful in comparing various spectral resolution methods and described how we generate realistic simulations that mimic many of the known properties of in vivo ^1^H-MRS data. Using simulated data generated with model resonances of a 12-metabolites basis set we have shown that ICA, without using any prior information about underlying metabolites, can identify the key structures underlying the data and resolve data into a linear mixture of components resembling the model resonances. The results show that ICA estimates correlate well with the ground-truth, comparable to the performances of LCModel in analyzing the same data. We demonstrate that ICA that collectively analyzes multiple spectra can be an effective tool in comparing multiple spectra.
